# RBMMMDA: predicting multiple types of disease-microRNA associations

**DOI:** 10.1038/srep13877

**Published:** 2015-09-08

**Authors:** Xing Chen, Chenggang Clarence Yan, Xiaotian Zhang, Zhaohui Li, Lixi Deng, Yongdong Zhang, Qionghai Dai

**Affiliations:** 1 National Center for Mathematics and Interdisciplinary Sciences, Chinese Academy of Sciences, Beijing, 100190, China; 2 Academy of Mathematics and Systems Science, Chinese Academy of Sciences, Beijing, 100190, China; 3 Department of Automation, Tsinghua University, Beijing, 100084, China; 4 School of Mechanical, Electrical & Information Engineering, Shandong University, Weihai, 264209, China; 5 School of Life Sciences, Tsinghua University, Beijing, 100084, China; 6 National Institute of Biological Sciences, Beijing, 102206, China; 7 Institute of Computing Technology, Chinese Academy of Sciences, Beijing, 100190, China; 8 University of Chinese Academy of Sciences, Beijing, 100049, China; 9 Key Lab of Intelligent Information Processing of Chinese Academy of Sciences, Institute of Computing Technology, Chinese Academy of Sciences, Beijing, 100190, China

## Abstract

Accumulating evidences have shown that plenty of miRNAs play fundamental and important roles in various biological processes and the deregulations of miRNAs are associated with a broad range of human diseases. However, the mechanisms underlying the dysregulations of miRNAs still have not been fully understood yet. All the previous computational approaches can only predict binary associations between diseases and miRNAs. Predicting multiple types of disease-miRNA associations can further broaden our understanding about the molecular basis of diseases in the level of miRNAs. In this study, the model of Restricted Boltzmann machine for multiple types of miRNA-disease association prediction (RBMMMDA) was developed to predict four different types of miRNA-disease associations. Based on this model, we could obtain not only new miRNA-disease associations, but also corresponding association types. To our knowledge, RBMMMDA is the first model which could computationally infer association types of miRNA-disease pairs. Leave-one-out cross validation was implemented for RBMMMDA and the AUC of 0.8606 demonstrated the reliable and effective performance of RBMMMDA. In the case studies about lung cancer, breast cancer, and global prediction for all the diseases simultaneously, 50, 42, and 45 out of top 100 predicted miRNA-disease association types were confirmed by recent biological experimental literatures, respectively.

MicroRNAs (miRNAs) are a highly abundant class of small (~22 nt), endogenous and evolutionarily conserved single-stranded non-coding RNAs that regulate the gene expression normally at post-transcriptional level by base pairing with their target mRNAs at 3′ untranslated regions (UTRs), causing mRNAs degradation or translational repression^1^,^2^,^3^,^4^,^5^,^6^. As is shown in many studies, the mature miRNAs are released from a much longer RNA molecule through mainly three steps, including cropping of pri-miRNA Q1 the Drosha/DGCR8 heterodimer and producing a miRNA precursor called pre-miRNA^7^,^8^, exporting and producing a mature miRNA duplex^9^, dicing and degraded resulting in a fully functioning miRNA^2^,^10^,^11^. In 1993, lin-4, as the first miRNA, was discovered when Victor Ambros and colleagues performed a genetic screen to investigate defects in the temporal control of Caenorhabditis elegans (C. elegans) development^12^. Then, some years later, the second miRNA (let-7) was discovered^13^. After the discovery of these two well-known miRNAs, many laboratories have focused their research on these small non-coding RNAs (ncRNAs)^14^,^15^,^16^,^17^,^18^,^19^ and many miRNAs have been found in plants, green algae, viruses, and animals^20^. According to the miRNA database (miRBase) released in 2014, there has been more than 2588 mature miRNAs in humans.

In the past few years, increasing evidences suggest that miRNAs play significant roles in a variety of essential biological processes, such as cell cycle regulation, differentiation, development, metabolism, neuronal patterning, aging and so on^3^. MiRNAs often play main regulator roles by binding to their target messenger RNAs (mRNAs) based on complete or partial sequence complementarity to induce transcript degradation or translational repression, respectively^21^. It has been demonstrated that a single miRNA can bind to multiple mRNAs and a target gene can be targeted by multiple miRNAs^22^. In addition to the complex base pairing between the miRNA and the mRNA, multiple miRNAs can also synergistically regulate one or more pathways^23^,^24^,^25^. It has been observed that miRNA-related regulations are complicated and evolutionarily conserved^26^,^27^,^28^. Therefore, it is no surprise that accumulating evidences show that the deregulations of miRNAs are associated with a broad range of human diseases, such as cancer, neurological disorders, cardiovascular disorders and so on^29^. For example, miR-17–92 cluster is shown to be oncogenic and responsible for malignant lymphoma^30^,^31^. MiR-206 can slow down the progression of ALS by sensing motor neuron injury and the miR-206 deficiency in ALS mouse model accelerate disease progression^32^. The miR-1 is involved in heart development and deletion of miRNA-1–2 interrupts the regulation of cardiogenesis^33^,^34^. Especially, the alterations of miRNA expression are involved in the initiation, progression and metastasis of various types of human cancers^35^, such as breast cancer^36^, lung cancer^37^, prostate cancer^38^, colon cancer^39^ and ovarian cancer^40^. Meanwhile, new disease related-miRNAs evidenced by several lines of experimental literatures are constantly emerging. Moreover, miRNAs have become a class of potential biomarkers for detection, diagnosis, prognosis and therapy in various human complex diseases^41^,^42^,^43^. Therefore, the disease-related miRNAs identifications are critical for not only understanding the molecular mechanisms of diverse diseases, but also providing potential biomarkers in disease diagnosis, treatment, and prognosis, and potential drug targets in drug discovery and clinical treatment.

Although many researchers have devoted their efforts on miRNA-disease interactions identification, the mechanisms underlying the dysregulations of miRNAs still have not been fully understood yet. Especially, the dysregulations of miRNAs cause disease through various kinds of mechanism. In the Human MicroRNA Disease Database (HMDD)^44^, miRNA-disease associations were divided into four types according to the different supporting evidences, including miRNA-disease association data from the evidence of genetics, epigenetics, circulating miRNAs and miRNA-target interactions, respectively. Genetic alterations (for example, SNP or deletion) and epigenetic changes (such as CpG methylation at the promoter, and abnormal histone modification) may affect the transcription of the pri-miRNAs, causing aberrant expression level of the miRNAs and leading to diseases. For example, chronic lymphocytic leukemia resulted from the deletion and subsequent down-regulation of the miR-15 and miR-16 at 13q14 locus^45^, and methylation of the miR-200b promoter reduced its expression and was associated with metastasis or hormone receptor status in advanced breast cancer^46^. Nowadays, the circulating miRNAs are considered to be diagnostic biomarkers^47^. For example, let-7a and miR-16 were associated with the progression-free survival and overall survival of myelodysplastic syndrome (MDS) patients and could serve as noninvasive prognostic makers^48^. MiRNAs can bind to the UTR region of the mRNA and induce its degradation or repress its translation^49^. The dysregulations of the miRNA-target interactions leads to many kinds of disease, such as lung cancer induced by misregulation of miR-let-7 and its target KRAS^50^, Alzheimer’s disease associated with miR-103, miR-107 and their elevated target cofilin^51^. Furthermore, the same miRNA could be associated with the same disease based on different association types. For example, miR-137 inhibited the proliferation of lung cancer cells by targeting Cdc42 and Cdk6. Meanwhile, miR-137 was downregulated in lung cancer cells by DNA methylation^52^. Therefore, lung cancer-miR-137 association was classified into both epigenetic and miRNA-target interaction types.

In the recent years, based on the assumption that miRNAs which have similar functions are often associated with similar disease and vice versa, many approaches have been proposed to predict miRNA-disease associations. For example, Jiang *et al.*^53^ developed a computational model based on hypergeometric distribution to infer potential miRNA-diseases associations by integrating the miRNA functional interactions network, disease phenotype similarity network and the known phenome-microRNAome network. Only local similarity measure has been adopted, so the prediction accuracy is not satisfactory. Shi *et al.*^54^ predict miRNA-disease associations by taking advantage of the functional links between miRNA targets and disease genes in the protein-protein interaction network. Mørk *et al.*^21^ presented a miRPD method in which not only the disease-associated miRNAs but also the underlying related proteins were predicted by combining known and predicted miRNA-protein interactions with text mined protein-disease associations. However, above three methods both strongly rely on the predicted miRNA-target interactions with a high rate of false-positive and high false-negative results. Chen *et al.*^55^ developed the first global network-based method, RWRMDA, to predict novel miRNA-disease associations by implementing random walk on the miRNA functional similarity network, which doesn’t rely on predicted miRNA-target interactions. Then, Xuan *et al.*^56^ proposed a new method named HDMP to predict potential miRNAs associated with human disease based on weighted k most similar neighbors. In that study, the previous miRNA functional similarity calculation methods were improved by incorporating the information content of disease terms, disease phenotype similarity, and the information of miRNA family and cluster. RWRMDA and HDMP obtained a reliable performance for miRNA-disease association prediction, but they can’t be applied to the diseases without known related miRNAs. Furthermore, HDMP strongly rely on the selection of the number of neighbors considered in the model and it didn’t set different values of this parameter when different diseases were investigated. Based on the assumption that if miRNAs implicated in a specific tumor phenotype, their target genes will be aberrantly regulated, Xu *et al.*^4^ constructed a miRNA-target dysregulated network (MTDN) by integrating target prediction results and expression profiles data of miRNA and mRNA that in tumor and non-tumor tissues. Furthermore, feature vectors were extracted and support vector machine classifier was adopted to distinguish positive disease miRNAs from negative ones, respectively. However, this method needs the information of known negative disease-related miRNAs. By integrating the information of known miRNA-disease associations, disease-disease semantic similarity, and miRNA functional similarity, Chen *et al.*^57^ further developed a semi-supervised prediction method (RLSMDA) based on the assumption that functional similar miRNAs tend to be associated with similar diseases and the framework of regularized least squares. RLSMDA achieved excellent performance in the both cross validation and case studies about several important diseases. Specially, RLSMDA is a semi-supervised method, so it does not need negative samples. More importantly, RLSMDA demonstrated excellent predictive ability for diseases without any known related miRNAs.

However, all of these previous approaches can only predict binary associations between diseases and miRNAs. On the one hand, current rich information about different types of miRNA-disease associations has not been well exploited for disease-related miRNA prediction. On the other hand, no previous computational methods could predict the types of disease-miRNA associations. Predicting the different types of disease-miRNA associations can further broaden our understanding about the molecular basis of diseases in the level of miRNAs. In this paper, we developed the model of Restricted Boltzmann machine for multiple types of miRNA-disease association prediction (RBMMMDA) to predict different types of miRNA-disease associations. Based on this model, we could obtain not only new miRNA-disease associations, but also corresponding association types. Restricted Boltzmann machine (RBM) has become the core of deep learning and successfully applied to solve various problems. Based on the following considerations, we chose RBM to predict multiple miRNA-disease associations in this work. Firstly, compared to previous work in predicting miRNA-disease associations outlined in the introduction section, RBM model could be used to predict multi-type associations and has shown its powerful performance in multi-type drug-target interaction prediction^58^ and neuroimaging data analysis^59^. Secondly, RBM provides a self-contained framework to obtain competitive classifiers directly and does not need to collect biological features and implement feature selection when classical methods such as SVM are adopted^60^. Thirdly, RBM could capture strong high-order non-linear correlations between the activities of features in the layer and therefore has demonstrated a good predict performance^58^,^60^,^61^. To our knowledge, RBMMMDA is the first model which could infer association types of miRNA-disease pairs on a large scale.

Leave-one-out cross validation (LOOCV) was implemented for RBMMMDA based on the known experimentally verified multiple types of miRNA-disease associations obtained from HMDD. As a result, the AUC of 0.8606 demonstrated the reliable and effective performance of RBMMMDA. Furthermore, RBMMMDA was evaluated by the case studies of lung cancer and breast cancer. Fifty and forty-two out of top 100 predicted miRNA-disease association types were confirmed by recent biological experimental literatures, respectively. Especially, RBMMMDA is a global approach, which can predict miRNA-disease association types for all the diseases simultaneously. Therefore, we applied RBMMMDA to all the diseases investigated in this study simultaneously and confirmed 45 out of top 100 predicted miRNA-disease association types. These confirmed associations were involved with as many as 10 important human complex diseases, such as breast cancer, hepatocellular cancer, non-small-cell lung cancer, and colorectal cancer. The excellent performance in the LOOCV and case studies fully demonstrated the potential value of RBMMMDA for the identification of miRNA-disease association types and the detection of human disease biomarkers.

## Results

### Performance evaluation

In this paper, we implemented LOOCV on the known multiple types of miRNA-disease associations obtained from HMDD to evaluate the predictive performance of RBMMMDA. Here, we considered all the diseases simultaneously to implement the global LOOCV. As for the parameters, we chose the learning rate as 0.01 and iterative number as 100 according to previous successful study of applying the idea of Restricted Boltzmann machine (RBM) into drug-target interactions prediction^58^. Specifically, each known miRNA-disease association was left out in turn as test association and other known multiple types of miRNA-disease associations were taken as seed associations. After that, RBMMMDA model was trained and predictive results were provided. Then this test miRNA-disease association was ranked relative to candidate associations which include all the miRNA-disease pairs that don’t have known experimental evidences. If the rank of the test miRNA-disease association exceeds the given threshold, the RBM model was considered to predict this miRNA-disease association correctly.

**Figure 1:**
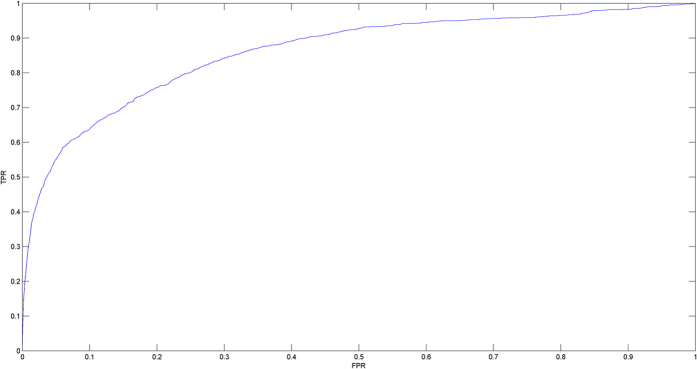
Performance evaluation of RBMMMDA in term of ROC curve and AUC based on LOOCV. As a result, RBMMMDA achieved a reliable AUC of 0.8606, demonstrating the reliable predictive ability of RBMMMDA. More importantly, RBMMMDA is the first method which could computationally predict the multiple types of miRNA-disease associations.

Finally, Receiver-Operating Characteristics (ROC) curve which plots true positive rate (TPR, sensitivity) versus false positive rate (FPR, 1-specificity) was drawn. Sensitivity refers to the percentage of the test miRNA-disease associations which are ranked higher than the given threshold. And specificity refers to the percentage of miRNA-disease associations that are below the threshold. Then the area under ROC curve (AUC) was calculated to evaluate the performance of RBMMMDA method. If AUC = 1, it means that the RBMMMDA method has perfect performance. And AUC = 0.5 indicates random performance. As a result, RBMMMDA achieved a reliable AUC of 0.8606 (See Fig. 1). Considering RBMMMDA is the first method to predict the multiple types of miRNA-disease associations, therefore there is no other method to implement performance comparisons. However, excellent predictive ability of RBMMMDA has been demonstrated based on the above LOOCV.

### Case studies

Researchers in the field of computational biology and machine learning are much concerned about overfit, which means the training error would keep decreasing steadily and the generalization error would start increasing instead of decreasing. In order to see whether RBM tends to overfit, case studies have been implemented to validate the multiple types of miRNA-disease associations in the prediction list. All the known multiple types of miRNA-disease associations in the gold standard dataset were used as training samples to predict potential miRNA-disease associations and their association types for several important diseases based on the model of RBMMMDA. Prediction results were verified based on recent biological experimental results to demonstrate the prediction ability of RBMMMDA.

Breast cancer is currently regarded as the most leading type of invasive cancer in women worldwide and it is estimated that there will be approximately 231,840 new cases of invasive breast cancer and 40,290 breast cancer deaths happen among US women in 2015^62^. The number of the affected people is still increasing, which has been predicted to reach nearly 3.2 million new cases per year by 2050^63^. Invasive breast cancer would occur in about one eighth of the women from the United States in her lifetime. Breast cancer can also be diagnosed in men, but with a much lower ratio than that in women^64^. The majority deaths of the breast cancer come from the developing countries, where most of the women are diagnosed in late stages^65^. Recently, growing evidence shows that several miRNAs are highly correlated with breast cancer and play important roles in the tumorigenesis of breast cancer. There are 176 miRNAs known to be related to the breast cancer in the golden standard dataset, and the associations of the breast cancer with related miRNA are categorized into four different subtypes according to the different supporting evidences. For example, mir-10b, which is up-regulated in metastatic breast carcinomas compared with the benign breast lesions, targets E-cadherin to promote tumor cell invasion, while mir-122 is down-regulated in breast cancer cells and functions to inhibit tumorigenesis of the cancer by targeting IGF1R^66^,^67^. This kind of miRNA and disease association is classified to be evidences from miRNA-target interaction. We implemented RBMMMDA to prioritize candidate miRNAs without the known relevance to breast cancer. As a result, among the top 10, 20 and 100 potential breast cancer-related miRNAs, 7, 13 and 42 miRNA-disease associations and their association type predications are supported by various biological experimental literatures, respectively (See Table 1 and Supplementary Table 1). It has been well-known that let-7 family mainly functions as tumor suppressors to inhibit breast cancer development and migration. Seven miRNAs from let-7 family has been ranked in top 10 predict list and five out of them has been confirmed by experimental literatures. For example, it has been confirmed that let-7i and let-7b can both inhibit the invasion of the breast cancer by targeting the oncogenes and tumor migration-related genes and induce tamoxifen sensitivity of the breast cancer by repressing the estrogen receptor α^68^,^69^,^70^,^71^,^72^; Down-regulation of let-7 g promotes breast cancer invasion by stimulating GAB2 and FN1 expression^73^; Androgen induced let-7a expression contributes to ER-, PR-, AR+ breast cancer pathogenesis^74^; Let-7f is a tumor-suppressor miRNA in breast cancer, which is induced by Aromatase inhibitors(Als) treatment to inhibit the aromatase gene and is also involved in low-dose metronomic (LDM) paclitaxel therapy by targeting Thrombospondin-1^75^,^76^. Both mir-193b and mir-221 has been ranked in the top 10 prediction list for breast cancer and confirmed by literatures. Mir-193b decreases in breast cancer cells, which allows the expression of its target genes DNAJC13 and RAB22A, and promotes breast cancer progression^77^. The plasma mir-221 is accumulated in breast cancer patients^78^, and may be a predictive biomarker for sensitivity to Neoadjuvant chemotherapy in patients with breast cancer^79^.

According to the American Cancer Society, the lung cancer is the most common cause of cancer deaths worldwide in both man and woman, which account for about 13% of all new cancers and 27% all cancer deaths, greater than the combination of colon, breast, and prostate cancer^80^. There are estimated 1.4 million deaths of lung cancer each year^80^,^81^,^82^,^83^. The most affected people come from North American, Europe and East Asia. Especially, lung cancer has become the first cause of death among people with malignant tumors in China and the registered lung cancer mortality rate has increased by 464.84%^84^. The five-year survival rate of lung cancer is much lower than many other leading cancers, such as breast cancer and prostate cancer, due to the fact that most lung cases are diagnosed at late stage^80^,^81^,^85^,^86^,^87^. So it’s important and urgent to study the mechanism of the tumorigenesis of lung cancer and screen for new biomarkers for early detection^80^,^81^,^88^,^89^,^90^. Recently, many miRNAs have been shown to play critical roles in lung cancer development and progression^91^,^92^,^93^. In our golden standard dataset, there are 52 lung cancer related microRNAs with various association types. For example, mir-101 is reduced in non-small cell lung cancer (NSCLC) and can suppress NSCLC development by targeting enhancer of Zeste Homolog2^94^, in contrast, the plasma miR-29c is significantly increased in NSCLC^95^. We further prioritize candidate miRNAs based on the scored calculated based on RBMMMDA. Half of top 100 potential lung cancer-related miRNAs and their association types are confirmed by several literatures. Especially, among the top 10 and top 20 prediction list, 90% of them have literature evidences (See Table 2 and Supplementary Table 2). In the top 10 potential related miRNAs, mir-34 family (a and b), which functions as tumor-suppressive miRNAs to induce apoptosis and inhibit proliferation in lung cancer cells by directly targeting TGFβR2 and Met, are inactivated by CpG methylation at their promoter region^96^,^97^,^98^,^99^,^100^,^101^,^102^,^103^; Also, mir-218, 133a and 143 are tumor-suppressors that play roles in inhibiting tumor cell invasion by targeting the tumorigenesis-related genes in lung cancer, such as N-cadherin, oncogenic receptors and so on^104^,^105^,^106^,^107^,^108^,^109^,^110^,^111^,^112^,^113^. There is also confirmed lung cancer-related miRNAs with the third association type. For example, sequence variants of mir-146a are associated with increased risk of NSCLC. Only mir-16 in the top 10 list currently has no supporting evidence.

RBMMMDA is a global ranking method, which could predict potential multiple association types of miRNA-disease pair for all the diseases simultaneously. Therefore, RBMMMDA was further applied to simultaneously rank all the candidate miRNA-disease associations. As a result, 45 of top 100 potential associations have experimental evidences (See Table 3 and Supplementary Table 3). Except for the breast and lung cancer, there are many other diseases involved in the top 100 list, such as Hepatocellular carcinoma, Stomach neoplasms, Melanoma, and so on. In the top 10 prediction list, except for 7 breast cancer-related miRNAs, the regulatory mechanism of which are mentioned above, other 3 miRNAs in the top 10 list all belong to mir-34 family. MiR-34a is shown to inhibit the growth and the metastasis of gastric cancer by directly targeting Met and PDGFR^114^. The CpG island methylation frequency of miR-34b in hepatocellular carcinoma cancer (HCC) is significantly higher in the tumor cells compared with that in adjacent non-tumor tissues, which may correlate to the inactivation of miR-34b in HCC^115^. MiR-34c is predicted to be related to prostatic Neoplasms, which currently has no experimental evidence. In the top 100 association, we can further observer that one miRNA may be related to one disease based on different association types, while it may also be associated with different diseases based on the same association type. For example, miR-34a, the methylation frequency of which is significantly higher in hepatocellular carcinoma than that in the non-tumor tissues, inhibits the invasion of both stomach neoplasms and hepatocellular carcinoma by targeting Met^114^,^115^,^116^,^117^.

In conclusions, fifty, forty-two, and forty-five out of top 100 predicted miRNA-disease association types for breast cancer, lung cancer, and global prediction have been confirmed by recent biological experimental literatures, respectively. All of these validation results demonstrate that RBMMMDA doesn’t have a tendency of overfit.

### Predicting novel multiple types of miRNA-disease associations

Here, after confirming the reliable performance of RBMMMDA in the framework of LOOCV and case studies, we further applied RBMMMDA to predict potential human miRNA-disease associations under the four different association types for all the diseases investigated in this paper. All the known multi-type miRNA-disease associations obtained from HMDD were used as training data. For all the 174 diseases, we publicly released the top 100 potential related miRNAs under four association types for each disease to facilitate experimental validation of human miRNA-disease associations. In the above case studies about breast cancer and lung cancer, most of the top 100 multiple types of miRNA-disease associations have been confirmed. Therefore it is anticipated that other potential multi-type miRNA-disease associations predicted by RBMMMDA could be validated by further biological experiments.

## Discussions

Identifying novel miRNA-disease associations and their corresponding association types is vitally important goal for biological development, which plays a critical role in the understanding of disease pathogenesis at the miRNA level. In this paper, we proposed the first computational method, RBMMMDA, to predict different types of miRNA-disease associations on a large scale based on known multiple types of miRNA-disease associations derived from HMDD. RBMMMDA approach can effectively encode multiple types of miRNA-disease associations by constructing an RBM model and can effectively predict different types of miRNA-disease associations, including genetics, epigenetics, circulating miRNAs and miRNA-target interactions, respectively. The performance of RBMMMDA was evaluated by implementing LOOCV on the known experimentally verified multiple types of miRNA-disease associations. The AUC score of 0.8606 demonstrated the reliable and effective performance of RBMMMDA. Moreover, we implemented case studies of breast cancer and lung cancer for further evaluations, in which fifty and forty-two out of top 100 predicted miRNA-disease association types were confirmed by recent biological experimental literatures, respectively. More importantly, RBMMMDA was applied to predict multiple types of miRNA-disease associations for all the disease simultaneously and forty-five out of top 100 results were confirmed. All of these show the reliable performance of RBMMMDA. It is anticipated that RBMMMDA could be an important and valuable computational tool for miRNA-disease association prediction and miRNA biomarker identification for human disease diagnosis, treatment, prognosis and prevention.

The reliable performance of RBMMMDA can largely be attributed to the combination of the following several factors. Firstly, RBMMMDA takes full advantage of known multiple types of miRNA-disease associations obtained from HMDD to implement predictions, which can further help us to understanding the molecular basis of diseases in the level of miRNAs under four different association types. Secondly, as far as we know, compared with previous methods that could only predict the binary associations between miRNAs and diseases, RBMMMDA is the first computational approach for multiple types of miRNA-disease association prediction, which can not only predict potential miRNA-disease associations, but also their corresponding association types. Finally, RBMMMDA could be applied to predict miRNA-disease association types for all diseases simultaneously.

Of course, some limitations also exist in the current version of RBMMMDA. Firstly, how to choose the appropriate parameter values in RBMMMDA is not still solved well. Secondly, the current version of RBMMMDA only takes advantage of the information of known multiple types of miRNA-disease associations. In the future, new biological information, such as the disease similarity information and miRNA functional similarity, could be also incorporated into our predictive model to further improve the performance of RBMMMDA. Currently, the RBM model only considered the connections between visible layer and hidden layer, the connections within the same layers is not allowed, thus how to integrate the data of disease similarity and miRNA similarity still require careful consideration. Thirdly, RBMMMDA is not applicable to the diseases without any known miRNA-disease association information. Finally, RBMMMDA may cause bias to miRNAs with more known associated diseases. In the future, with the existence of more available experimental verified multiple types of human miRNA-disease associations, the performance of RBMMMDA will further be improved.

## Methods

### Multiple types of miRNA-disease associations

In this paper, we downloaded the data of miRNA-disease associations from HMDD V2.0 (http://www.cuilab.cn/hmdd) constructed by Li *et al.*^44^, which provide a comprehensive resource of experimentally verified miRNA-disease associations and lays an important data fundamental for further miRNA-related computational research. The new version of database annotates miRNA-disease associations in more details, including miRNA-disease association data from miRNA-target interactions, circulation, epigenetics, and genetics. After getting rid of duplicate associations with the different evidences, we obtained 1680 distinct high-quality experimentally confirmed multi-type miRNA-disease associations about 174 diseases, 322 miRNAs and 4 different types of associations and used these miRNA-disease associations as training samples. Specifically, the data contains 682 miRNA-target interactions, 443 circulation, 199 epigenetics, and 356 genetics, respectively (see Supplementary Table 4). To our knowledge, the multiple types of miRNA-disease training samples used in this study have been the largest dataset until now. Considering training samples are incomplete and no previous computational models have been developed to solve this important problem, predicting multiple types of miRNA-disease associations is a difficult challenge. However, it is worth noting that RBMMMDA still obtained the reliable predictive performance in both LOOCV (AUC of 0.8606) and case studies about lung cancer, breast cancer, and global prediction for all the diseases simultaneously. More available associations obtained in the future would further improve the predictive performance of RBMMMDA.

### RBMMMDA

**Figure 2:**
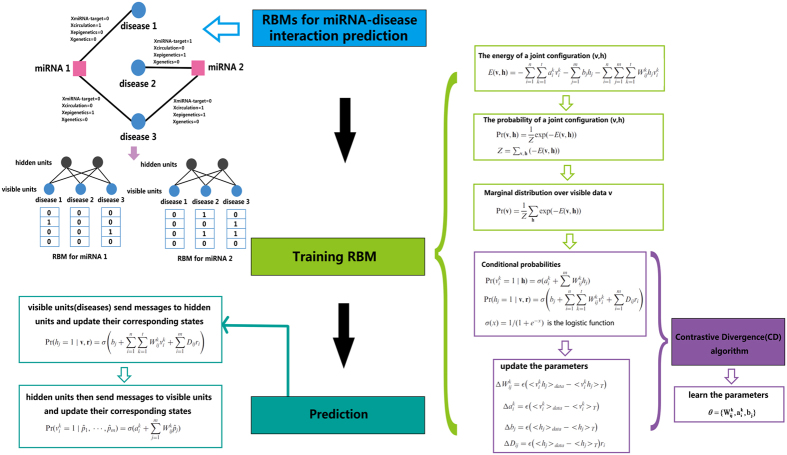
Flowchart of RBMMMDA, demonstrating the basic ideas of predicting multiple types of disease-miRNA association in the framework of RBM, which includes the basic there steps: constructing RBMs from a disease-miRNA interaction network; training RBM by CD algorithm; implementing prediction by computing conditional probabilities.

In this study, we developed the model of Restricted Boltzmann machine for multiple types of miRNA-disease association prediction (RBMMMDA) to predict different types of miRNA-disease associations (See Fig. 2, motivated by literature by Wang and Zeng^58^). Based on this model, we could obtain not only new miRNA-disease associations, but also their corresponding association types. RBM has been successfully applied to many important research fields^58^,^118^,^119^.

As shown in Fig. 2, RBM is a two-layer undirected graphical model consisting of layers of visible units and hidden units, respectively. In our RBM model, a visible unit is used to represent a disease. Hidden units represent unknown features describing miRNA-disease associations. In visible or hidden layer, there is no intra-layer connection. Furthermore, each visible unit is connected to all hidden units.

In the first step in Fig. 2, a simple example is provided to demonstrate how to construct RBMs from a multidimensional miRNA-disease interaction network. There are two miRNAs and three diseases which are included in this simple miRNA-disease network. Firstly, each miRNA-disease pair is associated with four binary variables, which indicates whether this miRNA-disease pair corresponds to this association type (i.e. the miRNA-disease associations from the evidences of miRNA-target interactions, circulation, epigenetics and genetics, respectively). Then, a particular RBM is constructed for each single miRNA. Here, two RBMs are constructed, and each RBM contains three visible units representing three diseases. The binary numbers inside rectangles represent the states of visible units to indicate whether a disease and a miRNA have a connection under each specific type. RBM model captures the existed multi-type connections between disease and miRNA pair in the multidimensional miRNA-disease interaction network to implement further prediction. In this example, we have known miRNA-disease associations for miRNA 1. Therefore, three diseases send messages to hidden units and update their states, and then the states of hidden units for miRNA 1 are obtained. After that, hidden units send messages again to visible units and update their states. Based on this idea, RBM is trained and potential multiple types of miRNA-disease associations are obtained.

### Training RBM

In an RBM, suppose that in total there are *n* visible units, *m* hidden units and *t* types of miRNA-disease associations encoded in a visible unit. **v** = (**v**_1_, …, **v**_***n***_) and **h** = (*h*_1_, …, *h*_*m*_) denotes the configuration of visible layer and hidden layer, respectively. Then (**v**, **h**) is a joint configuration of an RBM. In visible layer, the binary indicator vector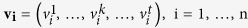, denotes the state of *i*-*th* visible unit. In hidden layer, *h*_*j*_, j = 1, …, m denotes the state of j-th hidden unit. Let 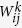 be the weight between visible variable 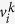 and hidden variable *h*_*j*_, and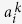, *b*_*j*_ denote bias weights of visible units and hidden units, respectively. To further formulate our RBM model, a binary indicator vector **r** = (*r*_1_, …, *r*_*m*_) is adopted, in which *r*_*j*_ = 1 if there exists a known disease-miRNA interaction between the input miRNA and the j-th disease, and *r*_*j*_ =0 otherwise. And *D*_*ij*_ is a parameter describing the effect of **r** on **h**.

Then the energy of a joint configuration (**v**, **h**) can be defined by


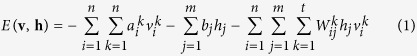


Then the probability of a joint configuration can be defined by


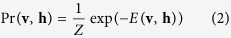


where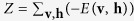 is called the normalizing constant or partition function. Then we can get the marginal distribution over all visible data **v** by summing all possible configurations of **h**.


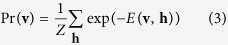


According to equation (3), we can get the probability distribution over input data. In visible layer or hidden layer, there is no intra-layer connection, so we can define the following conditional probabilities:


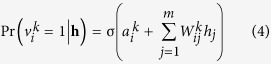



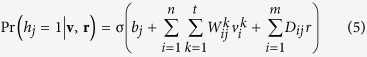


where *σ*(x) = 1/(1 + e^−*x*^) is the logistic function.

However, we do not know the values of many parameters, such as 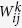, 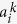, *b*_*j*_, *D*_*ij*_. Therefore, a mean-field version of the Contrastive Divergence (CD) algorithm is adopted here to train RBM and obtain the values of various parameters. In the CD algorithm, we use the following procedure in each training pass to incrementally adjust the weights and bias to maximize the likelihood of visible data with respect to the parameters 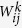, 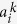, *b*_*j*_ and *D*_*ij*_.


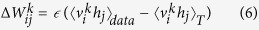



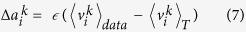



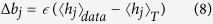



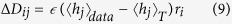


where 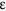 is the learning rate, 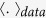 denotes an average value over all input data for each update and 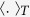 denotes the average value over T mean-field iteration. Based on CD algorithm, the parameters of RBM model are obtained. Therefore, we can use this RBM model to implement prediction.

### Prediction and Implementation

We can compute the following conditional probabilities after one mean-field iteration to predict the unknown interactions between disease and miRNA pair.


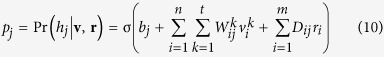



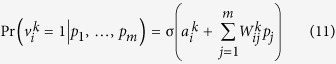


Because there is no intra-layer connection between any pair of visible or hidden units, once given the input data, we can get the state of hidden units according to equation (10). Then, use equation (11) we can get the probability distribution of visible units as our final prediction.

We implemented the whole algorithm in Java, and used the jaRBM package. In order to implement the algorithm, we need to initialize some variables. In this study, according to previous successful study of applying RBM to potential drug-target interactions prediction^58^, we set the number of hidden units m = 100, learning rate *ε* = 0.01, and chose Gaussian distribution with standard derivation of 0.1 to initialize 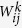, *h*_*j*_, 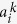 and *D*_*ij*_. As for other parameters, we used the default values defined in jaRBM package.

### Webserver of RBMMMDA

In addition, we built a web server which can implements the prediction function of RBMMMDA. This web server is freely available at http://42.120.43.172/RBMMMDA/. This web server enables the prediction of multiple types of miRNA-disease associations based on RBMMMDA method. When visitors choose a specific disease, potential miRNA associated with this disease based on various association types would be provided. The final prediction results would be shown in a table, where the miRNA name, association type, and potential association probability would be included.

## Additional Information

**How to cite this article**: Chen, X. *et al.* RBMMMDA: predicting multiple types of disease-microRNA associations. *Sci. Rep.* **5**, 13877; doi: 10.1038/srep13877 (2015).

## Supplementary Material

Supplementary material

Supplementary Table 1

Supplementary Table 2

Supplementary Table 3

Supplementary Table 4

## Figures and Tables

**Table 1:** We implemented RBMMMDA to prioritize candidate miRNAs without the known relevance to breast cancer.

miRNA	Type	Evidence (PMID)
hsa-let-7i	1	24662829; 21826373
hsa-let-7d	1	
hsa-let-7b	1	21826373;24264599;23339187;22761738
hsa-let-7g	1	21868760
hsa-let-7a	1	24172884
hsa-let-7e	1	
hsa-mir-183	2	
hsa-let-7f	1	22407818;25552929
hsa-mir-193b	1	25550792;25213330;19701247;21512034;19684618
hsa-mir-221	2	25009660;22156446
hsa-mir-92a	4	
hsa-mir-18a	2	24694649;23705859
hsa-mir-216a	2	
hsa-mir-138	1	25339353;20332227
hsa-let-7c	1	25388283
hsa-mir-187	1	20332227
hsa-mir-502	1	23291132;19789321;24677135
hsa-mir-376c	1	
hsa-mir-361	2	
hsa-mir-452	2	22353773

As a result, among the top 20 potential breast cancer-related miRNAs, 13 miRNA-disease associations and their association type predications are supported by various biological experimental literatures, respectively.

**Table 2:** We further prioritize candidate miRNAs based on the scored calculated based on RBMMMDA for lung cancer. Among the top 20 prediction list, 90% of them have literature evidences.

miRNA	Type	Evidence (PMID)
hsa-mir-34a	3	21383543;18719384
hsa-mir-218	1	21159652;24247270;24705471;
hsa-mir-34b	3	24130071;22047961;21383543
hsa-mir-127	3	24665010
hsa-mir-133a	1	25518741;24816813;22089643
hsa-mir-146a	4	25524943;25154761;24144839;21902575
hsa-mir-16	4	
hsa-mir-34a	1	25501507;25038915;24983493;
hsa-mir-143	1	25322940;25003638;24070896
hsa-mir-34b	1	23314612
hsa-mir-34c	3	24130071;22047961;21383543
hsa-mir-221	1	18246122;21042732;19962668;25151966
hsa-mir-182	1	25012722;24600991;23877371;21503569
hsa-mir-15a	1	25442346;24500260
hsa-mir-27a	1	25128483
hsa-mir-200a	1	23938385; 23708087
hsa-mir-9	3	24356455;24649145;22282464
hsa-mir-17	4	
hsa-mir-34c	1	23805317;22370637
hsa-mir-16	1	25435430;23954293

**Table 3:** RBMMMDA is a global ranking method, which could predict potential multiple association types of miRNA-disease pairs for all the diseases simultaneously.

Disease	miRNA	Type	Evidence (PMID)
Breast Neoplasms	hsa-let-7i	1	24662829;21826373
Breast Neoplasms	hsa-let-7d	1	
Prostatic Neoplasms	hsa-mir-34c	3	
Breast Neoplasms	hsa-let-7b	1	21826373;23339187;24264599;22761738
Breast Neoplasms	hsa-let-7g	1	21868760
Breast Neoplasms	hsa-let-7a	1	24172884
Breast Neoplasms	hsa-let-7e	1	
Carcinoma, Hepatocellular	hsa-mir-34b	3	24704024
Breast Neoplasms	hsa-mir-183	2	
Stomach Neoplasms	hsa-mir-34a	1	24837198
Carcinoma, Hepatocellular	hsa-mir-34c	3	
Neoplasms	hsa-mir-145	1	24999188;24801908; 24690171;24642628
Breast Neoplasms	hsa-let-7f	1	22407818;25552929
Breast Neoplasms	hsa-mir-193b	1	25550792;25213330;19701247;21512034;19684618
Breast Neoplasms	hsa-mir-221	2	25009660;22156446
Breast Neoplasms	hsa-mir-92a	4	
Breast Neoplasms	hsa-mir-18a	2	24694649;23705859
Breast Neoplasms	hsa-mir-216a	2	
Carcinoma, Hepatocellular	hsa-mir-34a	3	24704024
Breast Neoplasms	hsa-mir-138	1	25339353;20332227;

Therefore, RBMMMDA was further applied to simultaneously rank all the candidate miRNA-disease associations. As a result, 13 of top 20 potential associations have experimental evidences.
