# Supplementary material for: RBMMMDA: predicting multiple types of disease-microRNA associations
Source: Sci Rep. 2015 Sep 8;5:13877. doi: 10.1038/srep13877 (PMC4561957; doi:10.1038/srep13877)
Supplement: Supplementary material [file srep13877-s1.pdf]

RBMMMDA: predicting multiple types of disease-microRNA associations

Xing Chen[1, 2,*,#], Chenggang Clarence Yan[3,#], Xiaotian Zhang[4], Zhaohui Li[5.6], Lixi Deng[7.8], Yongdong Zhang[9], Qionghai Dai[3]

[1]National Center for Mathematics and Interdisciplinary Sciences, Chinese Academy of Sciences, Beijing, 100190, China

[2]Academy of Mathematics and Systems Science, Chinese Academy of Sciences, Beijing, 100190, China

[3]Department of Automation, Tsinghua University, Beijing, 100084, China

[4]School of Mechanical, Electrical & Information Engineering, Shandong University, Weihai, 264209, China

[5]School of Life Sciences, Tsinghua University, Beijing, 100084, China

[6] National Institute of Biological Sciences, Beijing, 102206, China

[7] Institute of Computing Technology, Chinese Academy of Sciences, Beijing, 100190, China

[8]University of Chinese Academy of Sciences, Beijing, 100049, China

[9]Key Lab of Intelligent Information Processing of Chinese Academy of Sciences, Institute of Computing Technology, Chinese Academy of Sciences, Beijing, 100190, China

*Corresponding authors

#The authors wish it to be known that, in their opinion, the first two authors should be regarded as joint First Authors.

Email: xingchen@amss.ac.cn

Supplementary Information

Supplementary Table 1. We implemented RBMMMDA to prioritize candidate miRNAs without the known relevance to breast cancer. As a result, among the top 100 potential breast cancer-related miRNAs, 42 miRNA-disease associations and their association type predications are supported by various biological experimental literatures, respectively.

Supplementary Table 2. We implemented RBMMMDA to prioritize candidate miRNAs without the known relevance to lung cancer. As a result, among the top 100 potential breast cancer-related miRNAs, 50 miRNA-disease associations and their association type predications are supported by various biological experimental literatures, respectively.

Supplementary Table 3. RBMMMDA is a global ranking method, which could predict potential multiple association types of miRNA-disease pair for all the diseases simultaneously. Therefore, RBMMMDA was further applied to simultaneously rank all the candidate miRNA-disease associations. As a result, 45 of top 100 potential associations have experimental evidences

Supplementary Table 4. The recent version of miRNA-disease association dataset in HMDD was downloaded. The new version of database annotates miRNA-disease associations in more details, including miRNA-disease association data from miRNA-target interactions, circulation, epigenetics, and genetics. After getting rid of duplicate associations with the different evidences, we obtained 1680 distinct high-quality experimentally confirmed multi-type miRNA-disease associations about 174 diseases, 322 miRNAs and 4 different types of associations. Specifically, the data contains 682 miRNA-target interactions, 443 circulation, 199 epigenetics, and 356 genetics, respectively
